# Transcription factor *Sp9* is a negative regulator of D1-type MSN development

**DOI:** 10.1038/s41420-022-01088-0

**Published:** 2022-06-30

**Authors:** Zhenmeiyu Li, Zicong Shang, Mengge Sun, Xin Jiang, Yu Tian, Lin Yang, Ziwu Wang, Zihao Su, Guoping Liu, Xiaosu li, Yan You, Zhengang Yang, Zhejun Xu, Zhuangzhi Zhang

**Affiliations:** Institute of Pediatrics, Children’s Hospital of Fudan University, state Key Laboratory of Medical Neurobiology and MOE Frontiers Center for Brain Science, Institutes of Brain Science, Fudan University, 200032 Shanghai, China

**Keywords:** Cellular neuroscience, Development of the nervous system

## Abstract

The striatum is the main input structure of the basal ganglia, receiving information from the cortex and the thalamus and consisting of D1- and D2- medium spiny neurons (MSNs). D1-MSNs and D2-MSNs are essential for motor control and cognitive behaviors and have implications in Parkinson’s Disease. In the present study, we demonstrated that *Sp9*-positive progenitors produced both D1-MSNs and D2-MSNs and that *Sp9* expression was rapidly downregulated in postmitotic D1-MSNs. Furthermore, we found that sustained *Sp9* expression in lateral ganglionic eminence (LGE) progenitor cells and their descendants led to promoting D2-MSN identity and repressing D1-MSN identity during striatal development. As a result, sustained *Sp9* expression resulted in an imbalance between D1-MSNs and D2-MSNs in the mouse striatum. In addition, the fate-changed D2-like MSNs survived normally in adulthood. Taken together, our findings supported that *Sp9* was sufficient to promote D2-MSN identity and repress D1-MSN identity, and *Sp9* was a negative regulator of D1-MSN fate.

## Introduction

The mammalian striatum, a crucial structure in the motor system, can receive and integrate excitatory inputs from the cortex, then the striatal signals flow to the internal segment of the globus pallidus (GPi) and the substantia nigra pars reticulata (SNr), which make outputs back to the cortex to control brain functions, such as movement, reward, motivation and cognitive behaviors [1]. Medium spiny neurons (MSNs), constituting 90%–95% of all neurons in the striatum, are derived from the ventral lateral ganglionic eminence (vLGE) during the embryonic period [2, 3]. Neural stem cells from the germinal zone (GZ) in the vLGE proliferate and then migrate radially to the mantle zone (MZ), where they undergo terminal neuronal differentiation to become MSNs of the striatum [4–7]. The D1-MSNs (striatonigral projection neurons) express the neuropeptide substance P (TAC1) and dopamine D1 receptor (DRD1). The D2-MSNs (striatopallidal projection neurons) express the neuropeptide enkephalin (PENK) and dopamine D2 receptor (DRD2) [1, 8, 9]. These two subpopulations of MSNs participate in two efferent pathways that functionally oppose each other and provide a balanced output from the basal ganglia [10]. This particular balance is widely believed to be crucial for normal motor control [11]. Abnormality or damage to striatal function leads to a spectrum of pathologies, such as Parkinson’s disease and Huntington’s disease, in humans [12–15]. Although previous single-cell analysis of the mouse striatum revealed diverse striatal cells in the adult striatum and the spatial distribution of different MSNs in the early postnatal striatum [16–18], the development of D1-MSNs and D2-MSNs and the mechanism by which they are targeted and integrated into striatal circuits are not fully understood. Furthermore, whether the neural stem cell pool of the vLGE exhibits diverse states and lineage plasticity in the specific time window during brain development has not been ascertained. These unresolved issues impelled us to explore lineage-targeted transcriptomics of the LGE at the single-cell level to identify key molecular determinants of MSN specification.

In this study, we identified a number of genes that were differentially expressed in two subpopulations of MSNs and a series of transcription factors that were found to be essential to the fate determination of MSNs. We demonstrated that *Sp9*-positive progenitors were common precursor cells of D1-MSNs and D2-MSNs. In particular, *Sp9*-positive progenitors formed the bridge linking neural stem cells to MSNs. As indicated in our previous study, the transcription factor *Sp9* was involved in striatal development, serving as a pivotal transcription factor for the development of D2-MSNs. Notably, in this case, *Sp9*-null mice lose most D2-MSNs, whereas the D1-MSNs development is largely unaffected [19, 20]. Accordingly, during striatal development, *Sp9* expression is downregulated in D1-MSNs, and the high level of *Sp9* expression is limited to D2-MSNs. These findings raised the possibility that the downregulation of *Sp9* expression may be necessary for the differentiation and maturation of D1-MSNs. We hypothesized that if *Sp9* expression was maintained in LGE progenitor cells and their progeny, including D1-restricted cells (pre-D1- MSNs), the fate of pre-D1-MSNs may change into D2-MSNs. To test this hypothesis, we generated *Rosa-Sp9-OE/*+ mice and obtained conditional *Sp9*-overexpression mice by crossing the *Rosa-Sp9-OE/*+mice with *Cre* reporter mice. We found that *Sp9* overexpression led to a reasonable proportion of pre-D1-MSNs developing into D2-like MSNs. Moreover, the fate switching between D1-MSNs and D2-like MSNs was gradual, and these D2-like MSNs survived normally in the adult brain.

## Results

### Single-cell transcriptome analysis delineates cellular heterogeneity in the mouse lateral ganglionic eminence

**Fig. 1:**
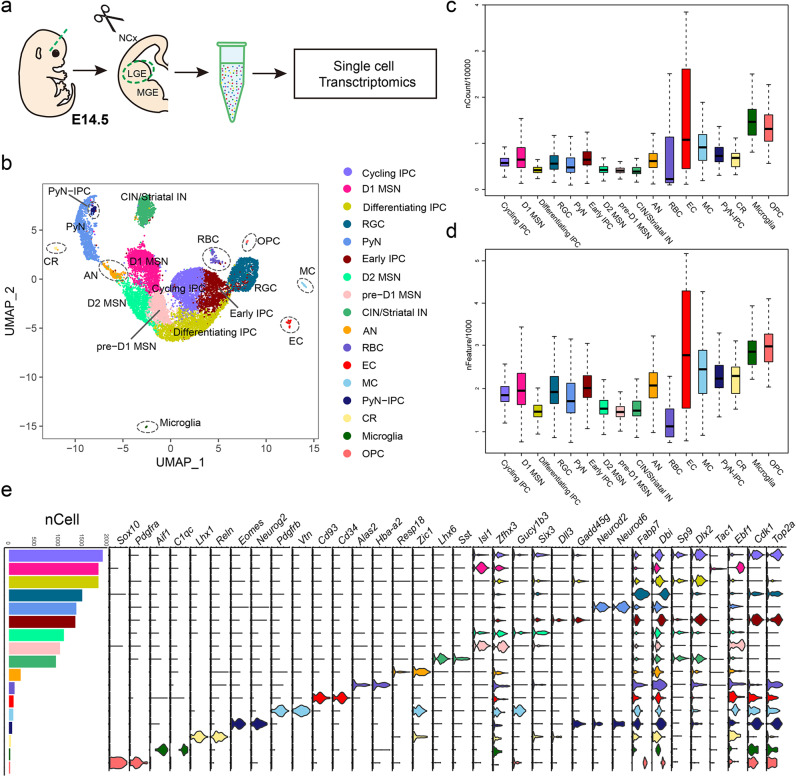
Single-cell transcriptome delineates mouse lateral ganglionic eminence cellular heterogeneity. **a** Schematic view of the experimental workflow. **b** Cellular composition of the lateral ganglionic eminence (LGE) was visualized using UMAP, and seventeen clusters were identified and annotated. Individual single-cell transcriptomes were colored on the basis of cluster identity. **c**, **d** Boxplots showed the number of unique molecular identifiers (UMIs, nCounts) per cluster (scale is in ten thousand) and the number of genes (nFeatures) detected per cluster (scale is in thousands). **e** Graph showed the number of cells per cluster. Violin plots depicted the expression of cell-type-specific marker genes for each cluster. Individual clusters was colored on the basis of cluster identity as shown in **b**. IN: interneurons, AN: amygdala neurons, RBC: red blood cells, EC: ependymal cells, MC: mural cells, CR: Cajal-Retzius cells.

The lateral ganglionic eminence in the ventral telencephalon is the primordium of the striatum. To systematically characterize the cell types in the LGE, we dissected the LGE from the mouse subpallium (eight total samples) at embryonic day 14.5 (E14.5), which is a crucial period in which striatal medium spiny neurons emerge, and we performed single-cell RNA sequencing (scRNA-seq) using 10X Genomics Chromium technology V3 [21] (Fig. 1a). A total of 17,555 single cells were harvested, and the transcriptome was profiled. Finally, 5587 unique molecular identifiers (UMIs) (median = 3837) and 1794 genes (median = 1620) per cell were detected (Fig. 1c, d). After quality control, a final data set of 14,001 cells was obtained. Unsupervised clustering using Uniform Manifold Approximation and Projection (UMAP) revealed seventeen clusters with distinct gene expression signatures (Fig. 1b). The principal cell types found within the LGE were radial glial cells (RGCs) (e.g., markers *Dbi* and *Fabp7*), early intermediate progenitors (early IPCs)(e.g., markers *Gadd45g* and *Dll3*), cycling intermediate progenitors (cycling IPCs) (e.g., markers *Top2a* and *Cdk1*), differentiating intermediate progenitors (differentiating IPCs) (e.g., markers *Dlx2* and *Sp9*), pre-D1-MSNs (e.g., markers *Zfp503* and *Isl1*), D1-MSNs (e.g., markers *Ebf1* and *Tac1*) and D2-MSNs (e.g., markers *Gucy1b3* and *Six3*) [18, 22] (Fig. 1e). The other cell clusters expressed the markers of projection pyramidal neurons (PyN) (e.g., markers *Neurod2* and *Neurod6*), interneurons (e.g., markers *Lhx6* and *Sst*), amygdala neurons (e.g., markers *Zic1* and *Resp18*), red blood cells (e.g., markers *Hba-a2* and *Alas2*), ependymal cells (e.g., markers *Cd34* and *Cd93*), mural cells (e.g., markers *Pdgfrb* and *Vtn*), PyN intermediate progenitors (PyN IPCs) (e.g., markers *Neurog2* and *Eomes*), Cajal-Retzius cells (e.g., markers *Reln* and *Lhx1*), Microglia (e.g., markers *C1qc* and *Aif1*) and OPC (e.g., markers *Sox10* and *Pdgfra*) [21] (Fig. 1e), which made up a small proportion of total cells within the LGE (Fig. 1b, e), and we therefore decided not to take these cell types into account in the following analyses.

### scRNA-seq reveals *Sp9*-positive progenitors are common progenitors of striatal MSNs

**Fig. 2:**
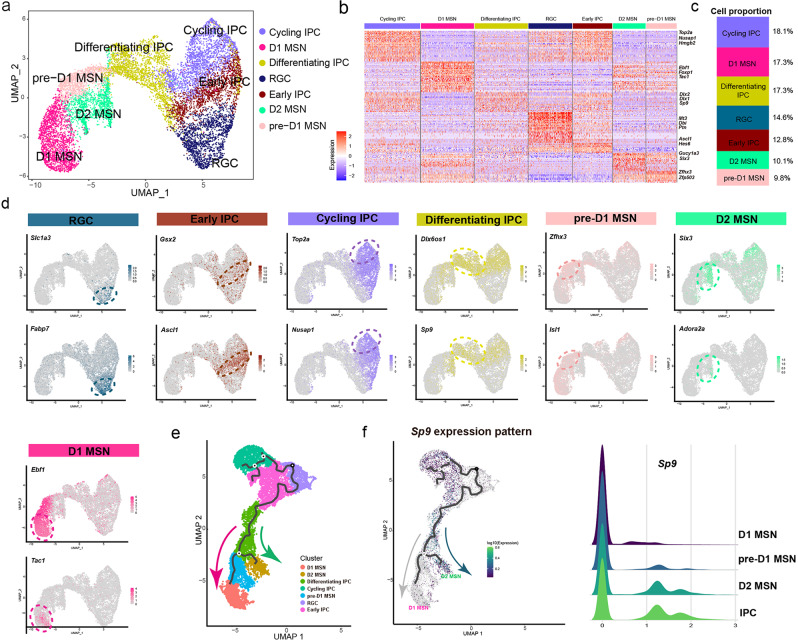
Identification of the developmental hierarchy of LGE cells. **a** The UMAP plot of LGE cells. **b** The heatmap of the LGE cells ordered by the UMAP showed in **a**. Each column represents expression in one cell, and each row represents expression of one gene. **c** The proportions of distinct clusters among total LGE cells. **d** UMAP plots of the expression levels of selected marker genes in the seven subpopulations. **e** Monocle3 revealed predicted lineage trajectories from RGC in the LGE; each point represents a cell and is colored on the basis of cluster identity. **f** *Sp9* expression pattern in the predicted lineage trajectories, and ridge plots shows *Sp9* expression distribution in the MSN Progenitor, pre-D1 MSN, D1 MSN, and D2 MSN.

Striatal medium spiny neurons and neuronal progenitors made up a majority of the total cell population (Fig. 1b, e). To directly investigate the development of MSNs, all irrelevant cells, including PyNs, interneurons, amygdala neurons, red blood cells, ependymal cells, mural cells, PyN IPCs, Cajal-Retzius cells, microglia and OPCs, were filtered for downstream clustering, resulting in seven clusters, including RGCs, IPCs (early IPCs, cycling IPCs, differentiating IPCs), pre-D1-MSNs, D1-MSNs and D2-MSNs (Fig. 2a). We showed seven clusters expressing RGCs markers (*Slc1a3* and *Fabp7*), early IPCs markers (*Gsx2, Ascl1*), cycling IPCs markers (*Top2a, Nusap1*), differentiating IPCs markers (*Dlx6os1, Sp9*), pre-D1-MSNs markers (*Zfhx3, Isl1*), D1-MSNs markers (*Ebf1* and *Tac1*), and D2-MSNs markers (*Six3*, *Adora2a*) (Fig. 2b, d). Among all these cells, 9.8% were pre-D1-MSNs, 17.3% were D1-MSNs and 10.1% were D2-MSNs (Fig. 2c).

To further investigate cell trajectories during MSN development, we utilized Monocle3, a statistical framework for inferring branching lineage assignments and developmental distances. Cell lineage development was predicted to start from radial glial cells passing through intermediate progenitors, after which two distinct trajectories were identified that led to either a D1-MSN or a D2-MSN fate (Fig. 2e). The trajectories showed that *Sp9*-positive progenitors were located between radial glial cells and MSNs, supporting the hypothesis that *Sp9*-positive progenitors serve as a transitional cell type and are common MSN progenitors. Notably, a series of transcription factors identified previously were expressed in these progenitors, including *Dlx1/2* [23], *Dlx5/6* [24, 25], and *Ascl1* [26, 27], indicating that they play vital roles in MSN differentiation. In this study, we showed the *Sp9* expression pattern in the trajectories, which revealed that *Sp9* was widely expressed in D2-MSNs and IPCs (early IPCs, cycling IPCs and differentiating IPCs), but was not expressed in pre-D1-MSNs or D1-MSNs (Fig. 2e, f). Taken together, the single-cell transcriptomics data support the hypothesis that *Sp9*-positive progenitor cells form a bridge, which contributes to the development of striatal MSNs, and the downregulation of *Sp9* may be essential for the development of D1-MSNs.

### *Sp9*-positive LGE progenitors give rise to both D1-MSNs and D2-MSNs but *Sp9* expression is downregulated in immature postmitotic D1-MSNs

**Fig. 3:**
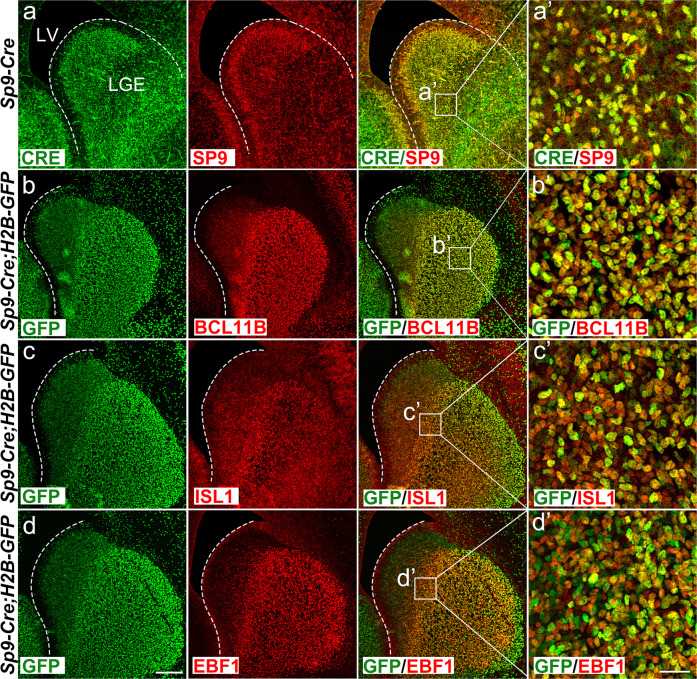
SP9^+^ LGE progenitors generate both D1-MSNs and D2-MSNs. **a** CRE/SP9 double immunostaining in the LGE of the *Sp9-Cre* mice at E16.5. **a’** Higher magnification images of CRE^+^/SP9^+^ cells in the LGE. **b** BCL11B/GFP double immunostaining in the *Sp9-Cre; H2B-GFP* LGE at E16.5. **b’** Higher magnification images of BCL11B ^+^/GFP^+^ cells in the LGE. **c** ISL1^+^/GFP^+^ double immunostaining in *Sp9-Cre; H2B-GFP* LGE at E16.5. **c’** Higher magnification images of ISL1^+^/GFP^+^ cells in the LGE. **d** EBF1^+^/GFP^+^ double immunostaining in *Sp9-Cre; H2B-GFP* LGE at E16.5. **d’** Higher magnification images of EBF1^+^/GFP^+^ cells in the LGE. Scale bars: 100 µm in **d** for **a**–**d**, and 25 µm in **d’** for **a’**–**d’**.

**Fig. 4:**
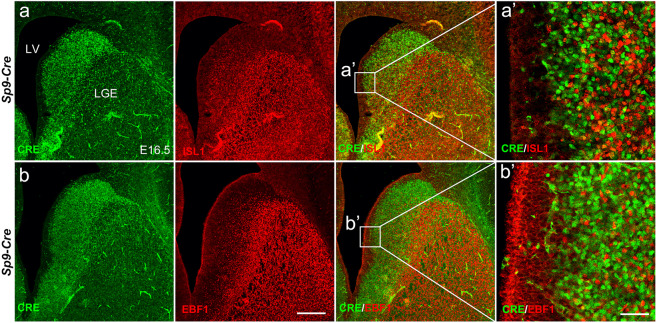
SP9 expression is downregulated in immature postmitotic D1-MSNs. **a** ISL1^+^/CRE^+^ double immunostaining in *Sp9-Cre* LGE at E16.5. **a’** Higher magnification images of the ISL1^+^/CRE^+^ cells in the LGE. **b** EBF1^+^/CRE^+^ double immunostaining in the *Sp9-Cre* LGE at E16.5. **b’** Higher magnification images of EBF1^+^/CRE^+^ cells in the LGE. Notably, nearly all ISL1^+^ or EBF1^+^ cells fail to be co-labeled with CRE (**a’** and **b’**). Scale bars: 200 µm in **b** for **a**–**b**, and 50 µm in **b’** for **a’**–**b’**.

Previously, we demonstrated that a subset of *Sp9*-positive cells in the LGE subventricular zone (SVZ) expressed the proneural gene *Ascl1*, suggesting that *Sp9* is expressed in LGE progenitors [20]. We also generated *Sp9-Cre* knock-in mice [20] and fate mapping at P30 using *Sp9-Cre; Rosa-YFP* mice showed that *Sp9*-positive progenitors generated all FOXP1 (a pan postmitotic MSN marker) positive cells in the LGE, indicating that, at the population level, *Sp9*-positive progenitors gave rise to both D1-MSNs and D2-MSNs. Our single-cell transcriptome analysis confirmed this initial finding, showing a clear developmental trajectory that *Sp9*-positive progenitors could transform into both D1-MSNs and D2-MSNs. In this study, we also proved that *Sp9-Cre* expression faithfully reflected the SP9 protein pattern, as virtually all *Sp9-Cre*-positive cells expressed the SP9 protein in the LGE at E16.5 (Fig. 3a-a’). Furthermore, immunostaining clearly showed that all BCL11B^+^, ISL1^+^ and EBF1^+^ cells were labeled with GFP in *Sp9-Cre; Rosa-H2b-GFP* LGE at E16.5 (Fig. 3b–d’). However, *Sp9* (*Sp9-cre*) expression was rapidly downregulated in the immature postmitotic D1-MSNs of the LGE SVZ while *Sp9* continued to be expressed in D2-MSNs, as only a few *Sp9-Cre*-positive cells expressed ISL1, and even fewer (or no) *Sp9-Cre*-positive cells expressed EBF1 (Fig. 4a, b’). These results revealed that *Sp9* was rapidly downregulated in immature postmitotic D1-MSNs while it was expressed in common progenitors of D1-MSNs and D2-MSNs, and *Sp9* was only maintained in D2-MSNs after MSN differentiation.

### Generation of *Rosa-Sp9-OE/* + mice

**Fig. 5:**
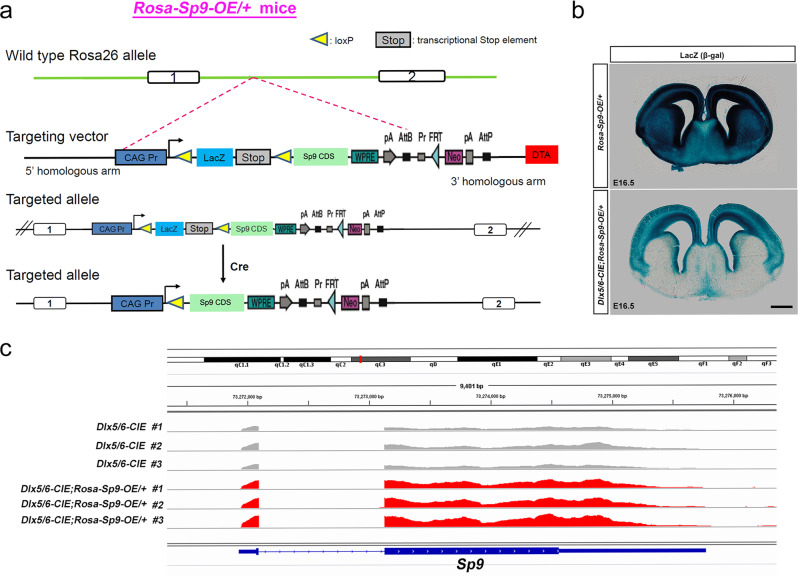
Generation of *Rosa-Sp9-OE/* *+* mice. **a** The construction strategy of generating mice with conditional *Sp9* overexpression (OE). **b** β-gal staining of *Dlx5/6-CIE* and *Dlx5/6-CIE; Rosa-Sp9-OE/* + brains at E16.5. **c** Bulk RNA-sequencing revealed higher *Sp9* expression levels (reads) in the *Dlx5/6-CIE; Rosa-Sp9-OE/* + LGE than in the control at E16.5. Scale bar: **b**, 500 µm.

The above observations inspired us to hypothesize that *Sp9* may play a critical role in the fate determination of D1-MSNs and D2-MSNs. Considering our previous study [20], we speculated that if *Sp9* was forcibly expressed in MSN progenitors and their descendants, more D2-MSNs and fewer D1-MSNs would be generated. Thus, we generated *Rosa-Sp9-OE/* + mice. Expression vector containing *CAG promoter-Flox-LacZ-STOP-Flox-Sp9 CDS-PolyA* was knocked into the *Rosa26* locus to get the conditional *Sp9*-overexpression mice (*Rosa-Sp9-OE/*+), the transcriptional stop signal maintained the *Sp9* gene in a transcriptionally silent state (Fig. 5a).

We obtained *Sp9*-overexpression mice by crossing the *Rosa-Sp9-OE/* + mice with *Cre* reporter mice. Beta-gal staining confirmed that the *Rosa-Sp9-OE/* + mice exhibited widespread beta-galactosidase (LacZ) activity in the whole brain (Fig. 5b). Since almost all striatal cells and the interneurons in the cortex originated from *Dlx5/6*-positive progenitors, *Dlx5/6-CIE*-induced *Sp9*-overexpression resulted in the whole striatum and partial cortex having no beta-galactosidase (LacZ) activity, indicating the integration of a functional transgenic cassette. To further verify that *Sp9* was overexpressed in *Dlx5/6-CIE; Rosa-Sp9-OE/* + mice, we performed bulk RNA-seq to compare the mRNA level of *Sp9* in *Dlx5/6-CIE; Rosa-Sp9-OE/* + LGE with that in control group (*Dlx5/6-CIE*). As expected, the peak of the *Sp9* gene in exon 1 and exon 2 in the experimental group was much higher than that in the control group (Fig. 5c). Thus far, we have proven that *Sp9* was conditionally overexpressed in the mice that we generated.

### *Sp9* overexpression downregulates the expression of D1-MSN-specific genes and upregulates the expression of D2-MSN-specific genes

**Fig. 6:**
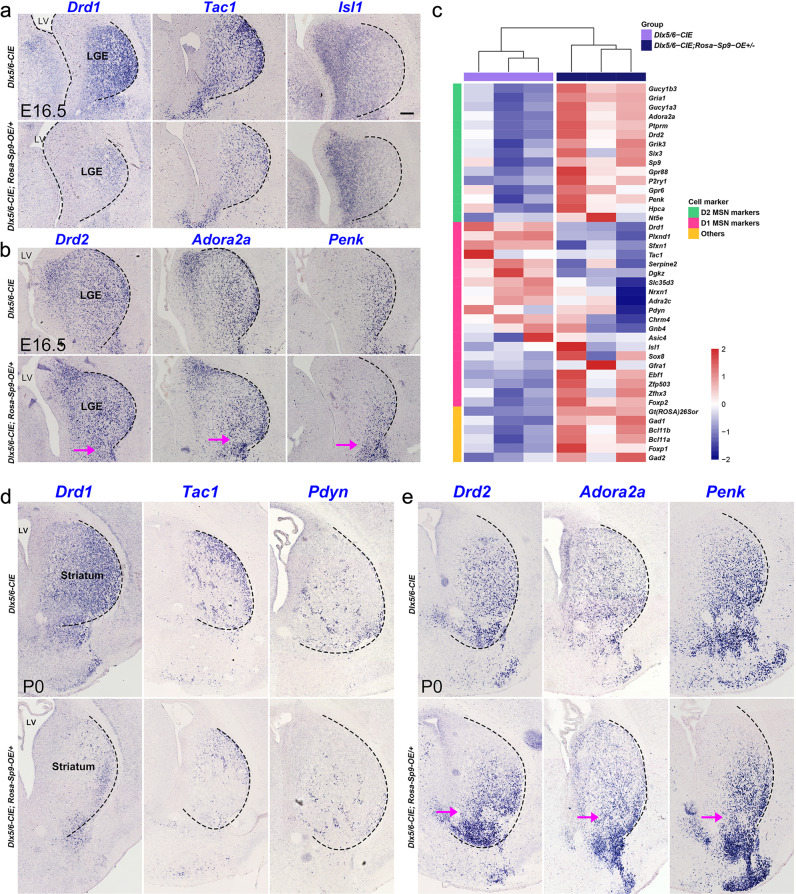
Sp9 overexpression represses the identity of D1-MSNs and promote the identity of D2-MSNs. **a** In situ hybridization staining showed a significant reduction in *Drd1* and *Tac1*, markers of D1-MSNs in the *Dlx5/6-CIE; Rosa-Sp9-OE/* + striatum, while the expression of *Isl1* was mostly unaffected in the *Dlx5/6-CIE; Rosa-Sp9-OE/* + striatum at E16.5. **b** In situ hybridization staining showed an increase in *Drd2*, *Adora2a* and *Penk*, markers of D2-MSNs in the *Dlx5/6-CIE; Rosa-Sp9-OE/* + striatum at E16.5. **c** Heatmap showing the expression levels of striatum-enriched genes (D1-MSNs enriched genes, D2-MSNs enriched genes and MSN-enriched genes) in E16.5 *Dlx5/6-CIE; Rosa-Sp9-OE/* + and *Dlx5/6-CIE* striatum (obtained from bulk RNA sequencing). **d** In situ hybridization showing that the expression of D1-MSN-enriched genes (*Drd1*, *Tac1* and *Pdyn*) were downregulated in P0 *Dlx5/6-CIE; Rosa-Sp9-OE/* + striatum. **e** In situ hybridization staining showed that D2-MSNs enriched genes (*Drd2, Adora2a and Penk*) were upregulated in P0 *Dlx5/6-CIE; Rosa-Sp9-OE/* + striatum. Magenta arrows indicate a significant up-regulation expression of *Drd2, Adora2a* and *Penk* in the ventral part of the striatum. Scale, 100 µm in **a** for **a**, **b** and **d**, **e**.

To examine the effect of *Sp9* overexpression on striatal MSN development, we bred *Rosa-Sp9-OE/* + mice with *Dlx5/6-CIE* (*Dlx5/6-Cre-IRES-EGFP*) mice [28, 29], which *Cre* activity was observed in the LGE progenitors of the developing brain. In situ hybridization showed a robust reduction in the expressions of *Drd1* and *Tac1*, markers of D1-MSNs [30–34], in the *Dlx5/6-CIE; Rosa-Sp9-OE/* + mice at E16.5, although we did not see the change of *Isl1* expression (Fig. 6a). In dramatic contrast to the decrease of D1-MSNs enriched genes, the expressions of *Drd2*, *Adora2a* and *Penk*, markers of D2-MSNs [19, 20, 22] were significantly increased (Fig. 6b), especially in the nucleus accumbens (NAc) and olfactory tubercle (OT). Moreover, bulk RNA-seq analysis further confirmed that a repertoire of D1-MSNs enriched genes, including *Drd1*, *Tac1*, *PlxnD1* and *Slc35d3*, was markedly decreased in *Dlx5/6-CIE; Rosa-Sp9-OE/* + (Fig. 6c) and *Sp9-Cre; Rosa-Sp9-OE/* + LGE at E16.5 (Table 1). In according with results from the in situ hybridization, D2-MSNs enriched genes, including *Drd2*, *Grik3*, *Gucy1a3* and *Penk*, were significantly increased in *Dlx5/6-CIE; Rosa-Sp9-OE/* + LGE at E16.5 (Fig. 6c).

Similarly, at P0, we obtained a consistent phenotype. We observed the increase of D2-MSNs enriched genes and the decrease of D1-MSNs enriched genes in *Dlx5/6-Cre; Rosa-Sp9-OE/* + mice than that in the control mice (*Dlx5/6-CIE*) (Fig. 6d, e). Altogether, these data provide strong evidences showing that *Sp9* overexpression is sufficient to repress the development of the D1-MSNs and promote the development of D2-MSNs.

### *Sp9* overexpression leads to a dramatic decrease in D1-MSNs and an increase in D2-MSNs in postnatal striatum

**Fig. 7:**
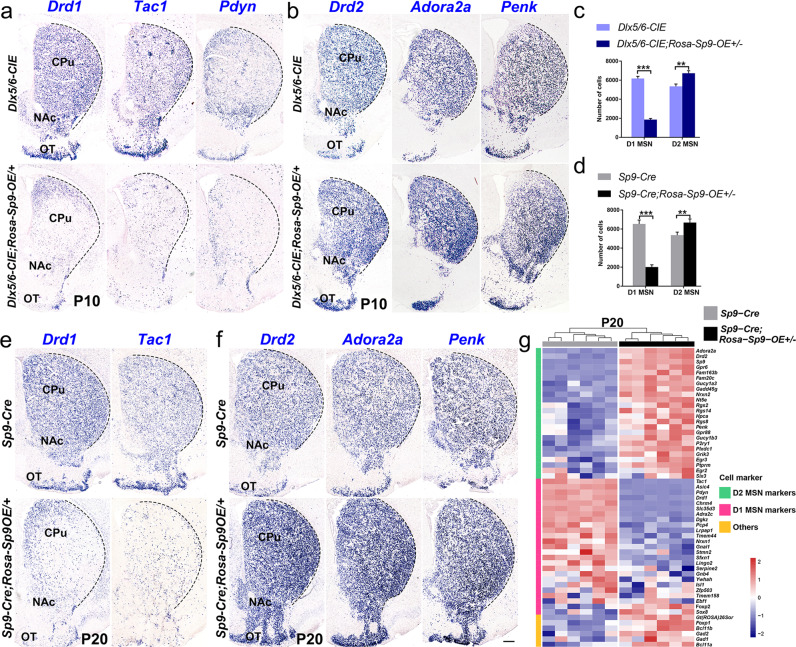
Sp9 overexpression decreases the number of D1-MSNs and increases the number of D2-MSNs. **a**–**c** In situ RNA hybridization of *Drd1*, *Tac1*, *Pdyn*, *Drd2, Adora2a* and *Penk* showing that the number of D1-MSNs was decreased by ~70% and that the number of D2-MSNs was increased by ~26% in the striatum of the *Dlx5/6-CIE; Rosa-Sp9-OE/* + mice compared to control mice at P10 (Student’s *t*-test, **p < 0.01, ***p < 0.001, *n* = 3, mean ± SEM). **d**–**f** In situ RNA hybridization showing that the number of D1-MSNs was decreased by ~70% and that the number of D2-MSNs was increased by ~25% in the striatum of the *Sp9-Cre; Rosa-Sp9-OE/* + mice compared with control mice at P20 (Student’s *t*-test, **p < 0.01, ***p < 0.001, *n* = 3, mean ± SEM). **g** The heatmap showing the expression level of MSN-enriched genes in the *Sp9-Cre; Rosa-Sp9-OE/* + and *Sp9-Cre* striatum at P20 (obtained by bulk RNA sequencing). The expression of the D1-MSN markers was greatly downregulated, and the expression of the D2-MSN markers was significantly upregulated. Scale bar: 500 µm in **f** for **a**, **b** and **e**, **f**.

To further detect the changes of D1-MSNs and D2-MSNs in *Sp9* overexpressing mice at the postnatal stage, we performed in situ hybridization in the *Dlx5/6-CIE; Rosa-Sp9-OE/* + mice at P10. As expected, the dramatic downregulation of the expression of D1-MSN-specific markers (*Drd1, Tac1* and *Pdyn*) was seen and the remaining signals were weak and scattered in the dorsal striatum. Conversely, the expression of D2-MSN-specific markers (*Drd2, Adora2a* and *Penk)* was increased significantly (Fig. 7a, b). These results indicated that most of the D1-MSNs did not accomplish the terminal differentiation in the *Dlx5/6-CIE; Rosa-Sp9-OE /* + mice at P10, this phenotype was even more remarkable than what we saw at P0. Consistently, there was an increase of D2-MSNs in the *Dlx5/6-CIE; Rosa-Sp9-OE/* + mice compared with the *Dlx5/6-CIE* mice, especially in the ventral and lateral striatum (NAc and OT). The statistical analysis showed that the number of D1-MSNs decreased by ~70%, while the number of D2-MSNs increased by ~26% in the *Dlx5/6-CIE; Rosa-Sp9-OE/* + mice compared with that in the control mice (*Dlx5/6-CIE*) (Fig. 7c).

We next sought to explore whether *Sp9-Cre; Rosa-Sp9-OE/* + showed a similar phenotype and we found that the number of D1-MSNs decreased by ~70%, while the number of D2-MSNs increased by ~23% at P20 when *Sp9* was forced to express by *Sp9-Cre* (Fig. 7d–f). Furthermore, bulk RNA-seq of the *Sp9-Cre; Rosa-Sp9-OE/* + striatum at P20 showed a consist result (Fig. 7g). Therefore, our results indicated that some D1-fate restricted MSNs did not differentiate normally and eventually transformed into D2-like MSNs in a slow manner in *Sp9* overexpression mice.

### Long-term survival of D2-like MSNs

**Fig. 8:**
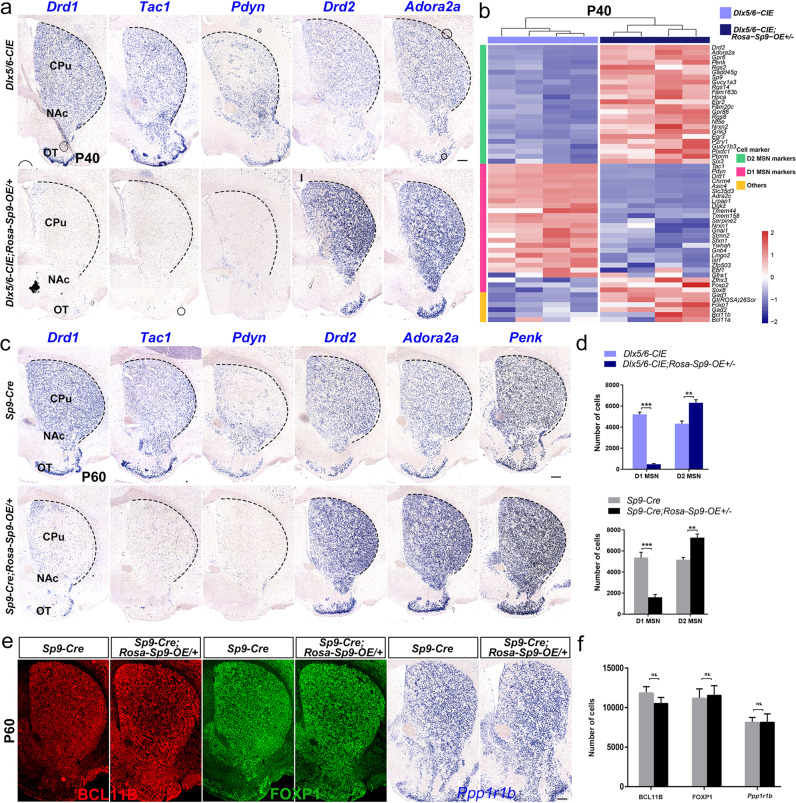
D2-like MSNs survive in the adult brain. **a**, **d** In situ RNA hybridization of *Drd1*, *Tac1*, *Pdyn*, *Drd2, Adora2a* and *Penk* showing that the number of D1-MSNs was decreased by ~91% and that the number of D2-MSNs was increased by ~46% in the striatum of the *Dlx5/6-CIE; Rosa-Sp9-OE/* + mice compared to the control mice at P40. **b** Heatmap showing the expression levels of MSN-enriched genes in the striatum of *Sp9-Cre; Rosa-Sp9-OE/*+ and *Sp9-Cre* striatum on P40 (obtained by bulk RNA sequencing). **c**, **d** In situ RNA hybridization showing that the number of D1-MSNs was decreased by ~71% and that the number of D2-MSNs was increased ~40% in the striatum of the *Sp9-Cre; Rosa-Sp9-OE/* + mice compared to control mice on P60, indicating that D2-like MSNs exhibit long-term survival. **e**, **f** The expression of BCL11B, FOXP1 and *Ppp1r1b* (three pan-striatal MSN markers) was similar in the *Sp9-Cre; Rosa-Sp9-OE/* + and control mice. (Student’s *t*-test, **p < 0.01, ***p < 0.001, *n* = 3, mean ± SEM). Scale bar: 250 µm in **e** for **a**, **c**, **e**.

As the results showed that some D1-fate restricted MSNs underwent fate change and transformed into D2-like MSNs in *Sp9* overexpressing mice. We further tested whether these D2-like MSNs can develop normally and survive to adulthood or even longer. To ascertain whether the D2-like MSNs exhibit long-term survival, we evaluated the phenotype of *Dlx5/6-CIE; Rosa-Sp9-OE/* + mice at P40 and *Sp9-Cre; Rosa-Sp9-OE/* + mice at P60 to observe the development of D1-MSNs and D2-MSNs. Our results showed that the expression of D2-MSNs enriched markers (*Drd2*, *Adora2a* and *Penk*) in *Sp9* overexpressing mice was still significantly increased (Fig. 8a–c), especially in the lateral striatum, NAc and OT. As previously reported [7], MSNs in the striatum were produced in the ventricles and migrated over a long distance to the lateral striatum, NAc and OT, indicating that the earliest produced cells were the farthest away from the ventricles. We speculated that the early produced D1-MSNs were the first to be transformed into D2-like MSNs in *Sp9* overexpressing mice. Notably, the expression of D1-MSNs enriched genes (*Drd1, Tac1* and *Pdyn*) in *Sp9* overexpressing mice was robustly decreased, we even could not detect the mRNA of D1-MSNs enriched genes in *Dlx5/6-CIE; Rosa-Sp9-OE/* + mice, a very few remaining *Drd1*-positive cells distributed in the medial part of the striatum, suggesting that these cells were born relatively later (Fig. 8a–c).

Our statistical analysis further showed that the number of D1-MSNs in the *Sp9* overexpressing mice was significantly decreased, and the number of D2-MSNs was greatly increased in adult (Fig. 8d). More D2-like MSNs were transformed at this moment than that at P20. To further explore that if the total number of striatal MSNs was changed, we performed staining of Foxp1, BCL11B and *Ppp1r1b*. Finally, we did not see the difference of the total number of MSNs between *Sp9-Cre; Rosa-Sp9-OE/* + mice and control mice (*Sp9-Cre*) at P60 (Fig. 8e, f), indicating that most of the D1-restricted MSNs switched to becoming D2-like MSNs in adulthood. In summary, our findings suggested that the D2-like MSNs in *Sp9* overexpressing mice can survive normally in adulthood.

## Discussion

The correct specification and differentiation of striatal projection neurons are important for normal brain function, and defects in these processes underlie motor and cognitive impairment. Abundant cell types are generated from radial glial cells directly or indirectly through transit-amplifying progenitors in the subpallium. The heterogeneity of subpallial progenitors has been established, but further study is needed. Our scRNA-seq data showed that neural stem cells and their progeny constituted a majority of the cells in the LGE at E14.5. It is unclear whether D1-MSNs and D2-MSNs are generated through distinct lineages or through the same type of progenitor cells. our pseudo-time analysis showed that radial glial cells gave rise to several types of progenitor cells, which generate D1-MSNs or D2-MSNs. *Sp9*-positive progenitor cells form a bridge linking the radial glial cells and MSNs in the striatal development, suggesting that D1-MSNs and D2-MSNs were generated by common intermediate progenitors.

In the present study, we have provided evidences that *Sp9* was highly expressed in MSN progenitors, and both D1-MSNs and D2-MSNs were generated by *Sp9*-positive LGE progenitors. Furthermore, *Sp9* expressed at a high level in D2-MSNs and a low level in D1-MSNs in postmitotic stage. These observations indicated that the downregulation of *Sp9* in MSN progenitors involved in D1-MSN differentiation, raising the possibility that Sp9 may play a vital role in the fate switching between D1-MSNs and D2-MSNs and encouraging us to investigate the role of *Sp9* in D1-MSNs. Our findings further demonstrated that *Sp9* overexpression led to increase of D2-MSNs at the expense of D1-MSNs, as indicated by the significantly upregulated expression of D2-MSNs related markers and the profoundly reduced expression of D1-MSNs related markers. Taken together, we proved that the transcription factor *Sp9* play a vital role in the fate switching between D1-MSNs and D2-MSNs, and it was a negative regulator of D1-MSN development.

Recent studies have showed that *Zfp503* was expressed in most, if not all, striatal MSNs. However, conditional knock out of *Zfp503* selectively affected the specification of D1-MSNs and canonical D1-MSN markers were absent or severely reduced while D2-MSN markers were ectopically increased [35, 36]. This outcome is very similar to our results showing that *Sp9* overexpression in D1-MSN lineage cells suppressed the expression of mature D1-MSNs markers (*Drd1*, *Tac1*, *Pdyn* and *Chrm4*) and promoted the expression of mature D2-MSNs markers (*Drd2*, *Adora2a*, *Penk* and *Gpr6*). Notably, the expression of a majority of canonical D1-MSNs markers was severely reduced and that most of D2-MSNs markers, except *Gpr6* and *Arpp21*, was greatly increased at the embryonic stage in the *Zfp503* knock out mice [35, 36]. We observed that in the *Sp9* overexpressing mice, the increase of D2-MSNs markers was very mild at the embryonic stage but dramatic at postnatal stage, especially after P10. These results indicated that the D1-MSNs fate was not fully established as shown by the fact that only a limited expression of D1-MSNs enriched genes was changed. *Zfp503* may have cooperated with *Sp9* to regulate D1-MSNs fate determination, probably *Zfp503* directly repressed *Sp9* expression in D1-MSNs. In the other case, *Zfp503* knockout mice showed that *Six3*, a *Sp9* downstream gene in D2-MSNs development, was significantly increased in D1-MSNs where *Six3* was typically not expressed, indicating that *Zfp503* represses *Six3* expression in D1-MSNs [19]. *Six3* has been found to be specifically expressed in D2-MSNs and to regulate D2-MSN development [19, 22]. Thus, *Six3* and *Zfp503* are candidates for regulating striatal MSN specification. In addition, several transcription factors, such as *Ebf1*, *Isl1* and *Foxo1*, have been reported to regulate D1-MSN specification but show little or mild effects on D2-MSNs [31–33, 37–39]. In study on *Sp9* overexpressing mice, the expression of *Ebf1*, *Isl1* and some D2-MSNs enriched genes were not significantly changed at embryonic stage (data not shown). However, at the postnatal stage, *Ebf1* and *Isl1* expression were rapidly downregulated and the expression of D2-MSNs enriched genes was gradually increased. These results suggested that *Ebf1* and *Isl1* may antagonize the D2-MSN specification by repressing effects of *Sp9* at embryonic stage.

Previous studies have confirmed that the pathogenesis of Parkinson’s disease and Huntington’s disease is related to the abnormal function of two projection loops in the striatum. Although functional studies have revealed distinct roles for D1-MSNs and D2-MSNs, the production and specification of D1-MSNs and D2-MSNs remain largely uncharacterized. Therefore, studying the development of the basal ganglion loop and exploring its molecular mechanism may provide a theoretical basis for further understanding the occurrence and development of clinical neurodevelopmental diseases. Our findings lay the foundation for understanding the function of the striatum and the pathogenesis of neurodevelopmental diseases.

## Materials and methods

### Animals

All animal experiments described in this study were approved in accordance with institutional guidelines at Fudan University Shanghai Medical College. In this study, mice that conditionally overexpressed *Sp9* were generated by knocking in an expression vector containing *CAG promoter-Flox-LacZ-STOP-Flox-Sp9 CDS-PolyA* into the *Rosa26* locus. With this strategy, the SP9 protein was continuously expressed in CRE recombinase-positive cells. In mice failing to express CRE recombinase, this vector would cause β-galactosidase but not the SP9 protein to be ubiquitously expressed. We described *Sp9-Cre* knock-in mice, *Dlx5/6-CIE* mice and *Rosa-YFP* mice in our previous reports [20, 29, 40]. Mice with mixed C57BL/6J, 129S6, and CD1 backgrounds were obtained. The morning on which a vaginal plug was detected was designated as embryonic day 0.5 (E0.5) and the day of birth was recorded as postnatal day 0 (P0).

### Immunohistochemistry and histochemistry

Immunohistochemistry and 5-bromo-4-chloro-3-indoyl-D-galactopyranoside (X-gal) staining were performed as previously described [20, 41]. Tissues were sliced into to 12 or 20 μm thick sections. The following primary antibodies were used to perform immunofluorescence labeling: mouse anti-CRE (Millipore, 69050-3), rabbit anti-EBF1 (Merck, AB10523), rabbit anti-ISL1 (Abcam, ab20670), chicken anti-GFP (AVES Labs, GFP-1020), rat anti-BCL11B (Abcam, ab18465), rabbit anti-FOXP1 (Abcam, ab16645) and rabbit anti-SP9 (1:500) [20]. Alexa Fluor 488-, Cy3- or 647-conjugated secondary antibodies were purchased from Jackson ImmunoResearch.

### In situ RNA hybridization

Digoxigenin-labeled riboprobes were generated and performed in situ hybridization on 20 μm cryostat sections as previously described [20, 41]. The riboprobes were generated by the following primers:

*Drd2* Forward: CGGGAGCTGGAAGCCTCGA

*Drd2* Reverse: TGCAGGGTCAAGAGAAGGCCG

*Adora2a* Forward: ATGGGCTCCTCGGTGTACATCATG

*Adora2a* Reverse: TCAGGAAGGGGCAAACTCTGAAGAC

*Drd1* Forward: ATGGCTCCTAACACTTCTACCATGG

*Drd1* Reverse: TCAGGTTGAATGCTGTCCGCTGTG

*Tac1* Forward: CCCCTGAACGCACTATCTATTC

*Tac1* Reverse: TAGAGTCAAATACCGAAGTCTCAG

*Isl1* Forward: TACGGGATCAAATGCGCCAA

*Isl1* Reverse: ACTCAGTACTTTCCAGGGCG

*Ppp1r1b* Forward: CCAGACACCCCAAGAACG

*Ppp1r1b* Reverse: GTGCCCAGGGGAGAGAAT

### RNA-seq

RNA-sequencing (RNA-seq) was performed at several developmental stages: on E16.5 with LGE, on P20 and P40 with striatum (*n* > = 3 for each group). Tissues were carefully dissected from the brains of mice exhibiting conditional *Sp9* overexpression (*Dlx5/6-CIE; Rosa-Sp9-OE/*+ or *Sp9-Cre; Rosa*-*Sp9-OE/*+ mice) and respective littermate controls (*Dlx5/6-CIE* or *Sp9-Cre*). Total RNA was purified with a Mini RNA isolation kit (Zymo) followed by library generation according to the manufacturer’s protocol (Illumina TruSeq Stranded Total RNA Library Prep Kit with Ribo-Zero Mouse). Fragment size distribution was assessed using a Bioanalyzer 2100. The concentration of the libraries was measured using a Kapa library quantification kit. The purified libraries were sequenced on a HiSeq 4000 platform. Levels of gene expression were reported in fragments per kilobase of exon per million fragments mapped (FPKM). Significantly differentially expressed genes were defined on the basis of a false detection rate (FDR) < 0.05. Differentially expressed genes were shown in a heatmap calculated with R language software.

### Tissue processing for single-cell RNA sequencing (scRNA-seq)

Embryonic mouse (E14.5) brains were quickly collected and placed in HBSS. The LGE was dissected and incubated in 1 mg/ml papain in HBSS for 20 min at 37 °C. The tissues were gently dissociated into a single-cell suspension by pipetting. Cells were centrifuged and washed twice, filtered through Flowmi Tip 40 μM strainers, and resuspended in HBSS + 0.04% BSA. Cell viability was assessed by trypan blue exclusion, and the cell concentration was adjusted for targeted sequencing of 10,000 cells/sample using a Chromium droplet-based sequencing platform (10X Genomics) Single Cell 30 Reagent Kits v3 protocol to prepare the libraries following the manufacturer’s instructions. The libraries were generated according to the manufacturer’s instructions and sequenced on an Illumina Hiseq4000. The raw data were processed and analyzed as previously described [21].

### Image acquisition

An Olympus BX 51 microscope or an Olympus FV1000 confocal laser scanning microscope system was used to collect images. All images were taken of approximately similar sections across samples. *Z*-stack confocal images were reconstructed with FV10-ASW software. All images were merged and optimized using Adobe Photoshop software.

### Quantification and statistics

At least three animals (randomized and assessed in a blinded manner) were used to obtain statistically significant data in each experiment. Student’s *t*-test was performed for two-group comparisons, and one-way ANOVA followed by Tukey’s post-hoc test was performed for comparisons of more than two-groups. For quantification of *Drd1-* and *Drd2*-positive cells in the striatum on P10, P20, P40 and P60, four 20 μm thick coronal sections from the rostral, intermediate, and caudal levels of the striatum were analyzed (*n* = 3 mice per group). We counted all *Drd1-* and *Drd2*-positive cells in the striatum. Brain regions were identified using a mouse brain atlas, and sections equivalent to the following bregma coordinates were assessed (in mm): the most rostral section, 1.18; the most caudal section, −0.10. Data are presented as the number of *Drd1-* and *Drd2*-positive cells per section for each striatum. For quantification of FOXP1-, BCL11B- and *Ppp1r1b*-positive cells in the mouse striatum on P60, four 20 μm thick coronal sections from the rostral, intermediate, and caudal levels of the striatum were selected (*n* = 3 mice per group). We used ImageJ software to count the number of FOXP1-, BCL11B-, and *Ppp1r1b*-positive cells in each striatum. Brain regions were identified using the mouse brain atlas, and sections equivalent to the following bregma coordinates were assessed (in mm): the most rostral section, 1.18; the most caudal section, −0.10. Data are presented as the number of FOXP1-, BCL11B- and *Ppp1r1b*-positive cells per section for each striatum. The cut-off for determining significance was *p* < 0.05 (*p < 0.05, **p < 0.01, and ***p < 0.001). The results were presented as the means ± SEM.

## Supplementary information

Table 1

## Data Availability

Bulk RNA-Seq and scRNA-Seq data are available from the Gene Expression Omnibus (GEO) database under accession number GSE202551.
